# Developing a potent vaccine against *Helicobacter pylori*: critical considerations and challenges

**DOI:** 10.1017/erm.2024.19

**Published:** 2024-11-25

**Authors:** Faria Hasanzadeh Haghighi, Shaho Menbari, Roghayeh Mohammadzadeh, Abbas Pishdadian, Hadi Farsiani

**Affiliations:** 1 Department of Microbiology and Virology, School of Medicine, Mashhad University of Medical Sciences, Mashhad, Iran; 2 Department of Medical Laboratory Sciences, Faculty of Paramedical Sciences, Kurdistan University of Medical Sciences, Sanandaj, Iran.; 3 Department of Immunology, School of Medicine, Zabol University of Medical Sciences, Zabol, Iran; 4 Antimicrobial Resistance Research Center, Mashhad University of Medical Sciences, Mashhad, Iran

**Keywords:** adjuvants, animal models, antigens, delivery system, *Helicobacter pylori* vaccines

## Abstract

*Helicobacter pylori* (*H. pylori)* is closely associated with gastric cancer and peptic ulcers. The effectiveness of antibiotic treatment against *H. pylori* is diminished by the emergence of drug-resistant strains, side effects, high cost and reinfections. Given the circumstances, it is imperative to develop a potent vaccination targeting *H. pylori.* Understanding *H. pylori*’s pathogenicity and the host’s immune response is essential to developing a vaccine. Furthermore, vaccine evaluation necessitates the careful selection of design formulation. This review article aims to provide a concise overview of the considerations involved in selecting the optimal antigen, adjuvant, vaccine delivery system and laboratory animal model for vaccine formulation. Furthermore, we will discuss some significant obstacles in the realm of developing a potent vaccination against *H. pylori.*

## Introduction

*H. pylori* is a helical and partially oxygen-dependent bacteria that can endure in the stomach and establish a permanent presence. The incidence of *H. pylori* infection exhibits significant disparity among countries, with rates as high as 80% in African nations and above 60% in Latin American countries (Ref. 1). Economic development, education level and sanitary conditions all have an impact on the variation in *H. pylori* infection prevalence (Ref. 2). Research has indicated that the primary variables contributing to the transmission of *H. pylori* during childhood are living in a crowded household, having a low socioeconomic position and having parents, particularly mothers, who are infected with *H. pylori* (Ref. 3). The primary modes of transmission for this infection are oral–oral, fecal–oral and gastro–oral routes (Ref. 4). Transmission by raw chicken flesh is another recently studied route of infection (Refs. 5, 6). A complex interaction of host, bacterial and environmental factors mediates the clinical consequences of *H. pylori* infections (Ref. 7). Possible consequences include gastritis, ulcers in the digestive tract, lymphoproliferative gastric lymphoma and even stomach cancer (Ref. 8). In addition, *H. pylori* is responsible for extra-gastrointestinal diseases, such as skin disorders, kidney illnesses, allergy symptoms, metabolic syndrome, ischemic cardiovascular disease and autoimmune diseases (Ref. 9). At present, there are four main first-line treatment regimens for *H. pylori*: clarithromycin-containing triple therapy, concurrent therapy, sequential therapy and bismuth quadruple therapy. The recommended initial treatment is quadruple therapy (Ref. 10). It is possible for probiotics to improve intestinal microecology and overall health through their anti-inflammatory and antioxidant processes; nevertheless, they are not capable of increasing the pace at which *H. pylori* infections are eradicated. Because of this, probiotic therapy can only be utilised as an additional therapy in order to lessen the number of adverse events that are associated with antibiotics (Ref. 11). Nevertheless, the eradication of *H. pylori* is becoming increasingly challenging due to various factors, including biofilm formation and resistance to antibiotics (Ref. 12). In addition, despite the successful elimination of bacteria, *H. pylori* infection can potentially recur, causing financial and psychological burdens for patients. Hence, it is imperative to prioritise the focus on vaccine development.

Despite the potential of the vaccine as a viable solution to achieve worldwide eradication of *H. pylori*, its development remains a formidable undertaking. The majority of research pertaining to this matter is still in its nascent phase and encounters significant obstacles, such as uncertainties surrounding *H. pylori*’s ability to evade the immune system and financial constraints (Refs. 13, 14). Subsequently, the quest for a vaccination against *H. pylori* has entered a phase of swift advancement. Multiple *H. pylori* vaccines have been subjected to ongoing or concluded clinical trials. The primary obstacles to the development of an *H. pylori* vaccine encompass the absence of sophisticated vaccine candidates (Refs. 13, 14), *H. pylori*’s immune evasion tactics (Ref. 15), restricted efficacy, insufficient animal models (Ref. 16) and the financial and adherence aspects (Ref. 17).

This review article seeks to offer a succinct summary of the factors to be taken into account when choosing the most suitable antigens, adjuvants, vaccine delivery systems, route of administration, laboratory animal models and the associated obstacles. Moreover, we will examine other substantial challenges in the field of establishing an efficacious vaccination for *H. pylori.*

### Vaccination against *H. pylori*, yes or not?

Considering that almost 30 years have passed since the initial vaccine against *H. pylori* underwent a clinical trial, and no further progress has been made, it prompts the question of whether immunisation against this bacterium should be pursued or not. If we persist in following this course of action, what are the impediments, and what strategies may we employ to enhance our accomplishments?

The development of a vaccine against *H. pylori* has been challenging, and there are currently only a few vaccines in phase I clinical trials (Refs. 14, 18, 19). In addition, some progress has been made in the production of an efficient vaccine against *H. pylori*, with a recent phase III clinical trial reporting good prophylactic aspects for an oral vaccine (Ref. 20). Vaccination against *H. pylori* might have either positive or negative outcomes. The potential risks of an *H. pylori* vaccine include the possibility of adverse effects for conditions that are inversely associated with *H. pylori* prevalence in worldwide populations, as *H. pylori* eradication may have unintended consequences (Ref. 18). Additionally, the limited protection generated in animal models raises concerns about the effectiveness of the vaccine in providing complete immunity (Ref. 13). Furthermore, the use of antibiotics in current *H. pylori* eradication therapies has drawbacks, such as limited compliance, adverse reactions and the risk of bacterial antibiotic resistance development (Ref. 21). Therefore, the potential risks of *H. pylori* vaccine development encompass not only the safety and efficacy of the vaccine itself but also the broader implications of *H. pylori* eradication and the limitations of current treatment options. Besides, vaccination has been shown to be effective in the prophylaxis and therapy of infectious diseases, and an *H. pylori* vaccine could protect against peptic ulcer disease and mucosa-associated lymphoid tissue lymphoma (Refs. 13, 22). Some vaccine formulations have shown a significant reduction in *H. pylori* colonisation in animal models, indicating the potential for disease prevention. Additionally, vaccination could limit the use of antibiotics for *H. pylori* treatment, potentially reducing adverse reactions and the development of antibiotic resistance (Refs. 14, 17). Overall, an effective *H. pylori* vaccine could provide significant benefits in terms of disease prevention, treatment and public health impact. Despite these challenges, vaccination against *H. pylori* is considered the only practical approach to large-scale elimination of the bacterium (Ref. 17).

### Current status of the *H. pylori* vaccine

Efforts by businesses and research institutions to create *H. pylori* vaccines in recent years have met with no results. Vaccines are now in their infancy, with the majority being in either phase I or preclinical development. Table 1 summarises the most important potential vaccines, adjuvants, animal models and immunological outcomes.

**Table 1.** A summary of the primary *Helicobacter pylori* vaccines published in the literature, including their compositional properties and immune response data

Vaccine	Antigen (s)	Type of vaccine	Prophylactic/Therapeutic	Route	Adjuvant (s)	Animal model	Immunological effects	Outcome	Stage	Ref.
*H. pylori* Hel 305	-	Whole cell	Prophylactic	Sublingual/Oral	mmCT	C57BL/6 mice	↑α4β7^+^CD4^+^ T cells, IFN-γ and IL–17A	↓Hp colonization	Preclinical	(38)
*H. pylori* SS1	-	Whole cell	Therapeutic	Oral	Chitosan particles	BALB/c mice	↑IL–12, IFN-γ, IL–2, IL–10, humoral, Th1 and Th2 responses	↓Gastritis and Hp colonization	Preclinical	(39)
*H. pylori*	-	Whole cell	Prophylactic	Intranasal/Oral	CpG-ODN	C57BL/6 mice	↑IgG2a and IFN-γ	Prevention 90%	Preclinical	(40)
*H. pylori* SS1	-	Whole cell	Prophylactic	Oral	α-GalCer	C57BL/6 mice	↑Intestinal and systemic Th1 responses, antibody, CD1d, IL–1R, IL–17R signaling	Prevention 70%, ↓inflammation	Preclinical	(21)
*H. pylori*	-	Whole cell	Therapeutic	Oral	LT (R192G)	-	↑Specific antibodies	Did not eradicate *H. pylori*	Phase I	(41)
*H. pylori*	-	Whole cell	Therapeutic	Oral	Chitosan particles	BALB/c mice	↑IFN, IL–12, IL–10, IL–4 ↓IgG2a/IgG1 ratio	Prevention 60%	Preclinical	(42)
pBudCE4.1 vector- *FlaA*	FlaA	Nucleic acid	-	Intramuscular	-	BALB/c mice	↑IgG, IgM, INF-γ, IL–2, IL–4 and IL–12	-	Preclinical	(43)
pcDNA3.-*cagW*–CS-NPs	CagW	Nucleic acid	Therapeutic	Intramuscular	Chitosan nanoparticles	BALB/c mice	↑IFN-γ, IL–2, IL–4, and IL–12, IgG and IgM	↓Hp colonization 100% of mice survived from challenge	Preclinical	(44)
pIRES2-*oipA*-IL(17–18–22)	OipA	Nucleic acid	Prophylactic	Intradermal	IL–17A, IL–18, IL–22, Foxp3	BALB/c mice	↑IgG1, IgG2, IgA ↑Th1 and Th17 response	Sterile immunity in IL–17-adjuvanted ↓4-log bacterial load in IL–22- adjuvanted	Preclinical	(45)
pcDNA3- *CagA-VacA-BabA*	CagA, VacA and BabA	Nucleic acid	Therapeutic	Intramuscular	PVP40	BALB/c mice	↑Apoptosis, T cell proliferation, TNF-α, Th1, Th2 and CD3^+^T cells activation ↓Infiltration FOXP3^+^ T cells	Suppress growth of GC	Preclinical	(46)
pVAX1-*pOipA*	OipA	Nucleic acid	Prophylactic	Intradermal	pIL–2 and pLTB	C57BL/6	↑IFN-γ, IL–2, IL–10, IL–12, IgG1 and IgG2a Shifting the immune response from a Th2 to a Th1	Sterile immunity in two mice (n = 10) ↓4-log bacterial load	Preclinical	(47)
CFAdE	UreA, UreB, Lpp20, HpaA,CagL	Epitope	Prophylactic	Oral	CTB, CFA, Polysaccharide adjuvant (PA)	BALB/c mice	↑IgG, sIgA, CD4^+^ Tcells	↓Hp colonization, Gastritis	Preclinical	(26)
FVpE	NAP, CagA, VacA, Urease	Epitope	Therapeutic	Oral	NAP, PA, LBP, chitosan	Mongolian gerbil	↑IgG, IgA, IFN-γ, IL–4, IL–17, CD4^+^ T cell	↓Hp colonization	Preclinical	(48)
HUepi	Urease, CagA, HpaA	Epitope	Therapeutic	Oral	LTB	BALB/c mice	↑CD4^+^ T cell Mucosal IgA, IgG	↓Hp colonization	Preclinical	(49)
CWAE	Urease, NAP, Hsp60 and HpaA	Epitope	Therapeutic	Oral	CTB, NAP, CFA, aluminium hydroxide	BALB/c mice	↑mixed CD4^+^ T cell response IgG, IgA (sIgA), IL–4, IFN-γ and IL–17	↓Gastritis, Hp colonization	Preclinical	(28)
Ty1033	UreA and UreB	Vector (*Salmonella enterica* Typhi)	Therapeutic	Oral	-	Human volunteers	No immune response to antigens	Couldn’t eradicate *H. pylori* infection, no serious adverse effects	Phase I	(31)
Ty21a- *UreA-UreB*	UreA and UreB	Vector (*S. enterica* Typhi)	Prophylactic	Oral	-	Human volunteers	Detected specific T helper cells in 69% (9 of 13)	Well tolerance, cannot satisfactory protection	Phase I	(50)
EGDeAB-MECU	UreB, FlaA, AlpB, SabA and HpaA	Vector (*Listeria monocytogenes*)	Therapeutic	Oral and Intravenous	-	BALB/c mice	↑IgG, IgA (sIgA), IL–4, IFN-γ, and IL–17	↓Hp colonization	Preclinical	(33)
UreB-LTB	UreB	Subunit	Prophylactic	Oral	LTB	children aged 6–15 years	↑IgG, IgA, sIgA, IL–4, IFN-γ and IL–2	Strong humoral and cellular immunity, can provide up to 3 years of continuous protection against *H. pylori* infection	Phase III	(20)
Multiantigen	VacA, CagA, NAP	Subunit	Prophylactic	Intramuscular	Aluminium hydroxide	Human volunteers	↑IgG, IgA, sIgA, IL–4, IFN-γ, IL–10, IL–17 and IL–2	Strong humoral and cellular immunity, cannot satisfactory protection	Phase I/II	(51)

Due to the continuous regeneration of the stomach mucosa and the acidic pH of the stomach, *H. pylori* is able to evade the body’s immunological response (Ref. 23). Also, complete eradication of *H. pylori* does not guarantee continuous safety. An *H. pylori* vaccination would decrease the occurrence and intensity of gastrointestinal diseases while also providing protection or large-scale elimination of the bacterium (Ref. 24). Choosing a viable technique for administering a preventative or therapeutic vaccine, along with efficient adjuvant and immunogenic bacterial antigens, is crucial (Ref. 25). Vaccines contain several antigens associated with vaccination, such as urease (UreB and UreA), vacuolating cytotoxin A (VacA), cytotoxin-associated gene A (CagA), neutrophil-activating protein A (NapA), *H. pylori* adhesin A (HpaA), blood group antigen-binding adhesion (BabA), hook-associated protein 2 homologue (FliD), outer membrane proteins (OMPs), heat shock protein A (HspA), gamma-glutamyl transpeptidase (GGT) and outer inflammatory protein A (OipA) (Ref. 15). The CFAdE (Ref. 26), CTB-HUUC (Ref. 27) and CWAE (Ref. 28) vaccines consist of antigens and adjuvants that contain epitopes specifically expressed on CD4^+^ and CD8^+^ cells. Mucosal adjuvants, such as cholera toxin (CT) and *Escherichia coli* enterotoxin, have been used to increase the immunogenicity of many vaccinations, including whole-cell, subunit and multiepitope vaccines (Ref. 29). Moreover, it is recommended to use intramuscular *H. pylori* subunit vaccines along with aluminum hydroxide adjuvants. Additionally, administering live vector vaccines, such as Salmonella, *Lactobacillus* and *Listeria monocytogenes*, that express *H. pylori* antigens orally can help improve long-lasting immunity (Refs. 30–33).

Vaccines are predominantly in the preclinical or phase I stages, exhibiting inconsistency and yielding varying outcomes. The findings of a phase III randomised trial, however, demonstrated that oral vaccinations containing recombinant UreB were both safe and efficacious in children (Refs. 14, 19, 20). *H. pylori* vaccinations proved ineffective in reducing microbial load and only offered limited immunity in smaller animals and people (Ref. 34). One of the best ways to stop malignant gastric tumors and other serious problems linked to *H. pylori* infection, though, would be to create a vaccine that targets the bacteria (Ref. 35). Especially in the context of antibiotic resistance, the development of vaccines could make a particularly significant contribution (Refs. 14, 24, 36). Potential candidates for the *H. pylori* vaccination are thoroughly reviewed in the references (Refs. 14, 36, 37).

### Host immune response against *H. pylori*

*H. pylori* can trigger a diverse range of immune responses, leading to chronic inflammation and infection in the stomach. Bacterial components, such as lipopolysaccharide (LPS), peptidoglycan, lipoteichoic acid, HspA, hypo-methylated CpG DNA and NapA, stimulate pattern recognition receptors, leading to the activation of many signal transduction pathways in gastric epithelial cells (Ref. 15). The intracellular signaling pathways involving mitogen-activated protein (MAP) kinases and NF-κB play a significant role in activating the c-fos and c-jun genes. This activation leads to a substantial increase in the production of proinflammatory cytokines, specifically IL-8 (Ref. 52). A recent study discovered a correlation between certain variations in the genes responsible for toll-like receptors (TLRs) 1, 2, 5 and 10 and an increased occurrence of *H. pylori* infection in a population from Turkey (Ref. 53). This discovery corroborates previous studies that have highlighted the significance of these pattern recognition receptors in the commencement of the infection (Refs. 54, 55). The conserved domain D1 is found in bacterial flagellins and is acknowledged by TLR5. It is noteworthy that *H. pylori* does not exhibit this domain. However, a recent study found that the CagL protein, which is a component of the type IV secretion system (T4SS), can activate TLR5 even in the absence of flagellins (Ref. 56). Furthermore, as reviewed in (Ref. 57), the T4SS plays a crucial role in facilitating the activity of CagA by delivering this pathogenic factor directly into the cells of the gastric epithelium.

At first, when the immune system is triggered, phagocytes are called upon, specifically in the stomach mucosa. Additional mechanisms include the production of targeted antibodies and the movement of activated CD4^+^ and CD8^+^ T cells to the stomach epithelium (Ref. 58). There is increasing evidence, suggesting that a T helper 1 (Th1) response, which stimulates inflammation, may arise (Ref. 59). Furthermore, inspection of *H. pylori* infection in adults discovered increased levels of IL-17, emphasising the significance of T helper 17 (Th17)-type cytokines in that particular context (Ref. 60). An interesting component of the effectiveness of the anti-*H. pylori* vaccine is its ability to stimulate the Th17 immune profile (Refs. 61, 62). *H. pylori* must decrease the activity, proliferation and clonal expansion of effector T cells (Th1 and Th17 subsets) in order to colonise successfully. GGT and VacA are two important virulence factors that destroy T-cell-mediated immunity. As a result, considering these two Th subsets and eliciting vaccination against GGT and VacA is critical to developing an effective vaccine (Ref. 63). Furthermore, interleukin-27 (IL-27) is a cytokine that plays a crucial role in determining the consequences of *H. pylori* infection. The latest investigation revealed that the levels of IL-27 are elevated in patients who are positive for *H. pylori* in comparison to those who are negative for *H. pylori.* Remarkably, this molecule was discovered to have a positive correlation with Th1 cytokine expression and a negative correlation with Th17 cytokine expression in both human serum and stomach mucosa (Ref. 64). When developing an anti-*H. pylori* vaccine, it is crucial to consider the role of IL-27 as it seems to have a substantial inhibitory impact on the Th17 profile.

Several studies evaluated cell- and antibody-mediated immunity in urease vaccine-induced *H. pylori* protection in mice. The research shows that vaccination with the urease antigen requires MHC class II-restricted, cell-mediated pathways to protect against *H. pylori* infection, not antibody responses. Cell-mediated immunity was essential to removing *H. pylori* in mice injected with urease vaccination and adjuvants (Refs. 65, 66). Post-*H. pylori* infection, gastrointestinal mucosa responses were dominated by CD4^+^ T cells, notably Th1 cells that produce interferon-gamma (IFN-γ) (Refs. 67, 68). In addition, *H. pylori* infection increased CD4^+^ T cells in rhesus monkey stomachs (Ref. 69). The main immunological responses seen were Th1 responses, typified by IL-2 and IFN-γ production, and proinflammatory cytokine responses. No T helper (Th2) response was observed (Ref. 69). Tregs suppress the immune system by releasing immunosuppressive cytokines like IL-10 and transforming growth factor-β (TGF-β) to manage the inflammatory response to *H. pylori* (Refs. 70, 71). In purposefully infected mice, Tregs decreased CD4^+^ T cell development, which may persist the infection (Refs. 72, 73). Conversely, mice without Treg cells had lower bacterial levels, increased Th1 responses and more severe gastritis (Ref. 72). According to accumulated evidence, the protective immunity that the *H. pylori* vaccination induces might not be an antibody-based response. Ermak *et al.* showed that the urease vaccination protected B-cell-deficient mice and wild-type mice (Ref. 66). A study found that B-cell-deficient (μMT) mice had better *H. pylori* eradication after 8 weeks of infection compared to wild-type mice (Ref. 74). However, investigations have shown that antibodies are essential for *H. pylori* eradication (Ref. 75). Targeted monoclonal antibodies can effectively inhibit urease (Ref. 76). Guo *et al.* created and tested the UreB vaccination on mice. This immunisation increased IgG and IgA antibody production, which blocked urease and reduced *H. pylori* in mice’s stomachs. Thus, increased antibodies may protect against *H. pylori* (Ref. 77).

**Figure 1.**
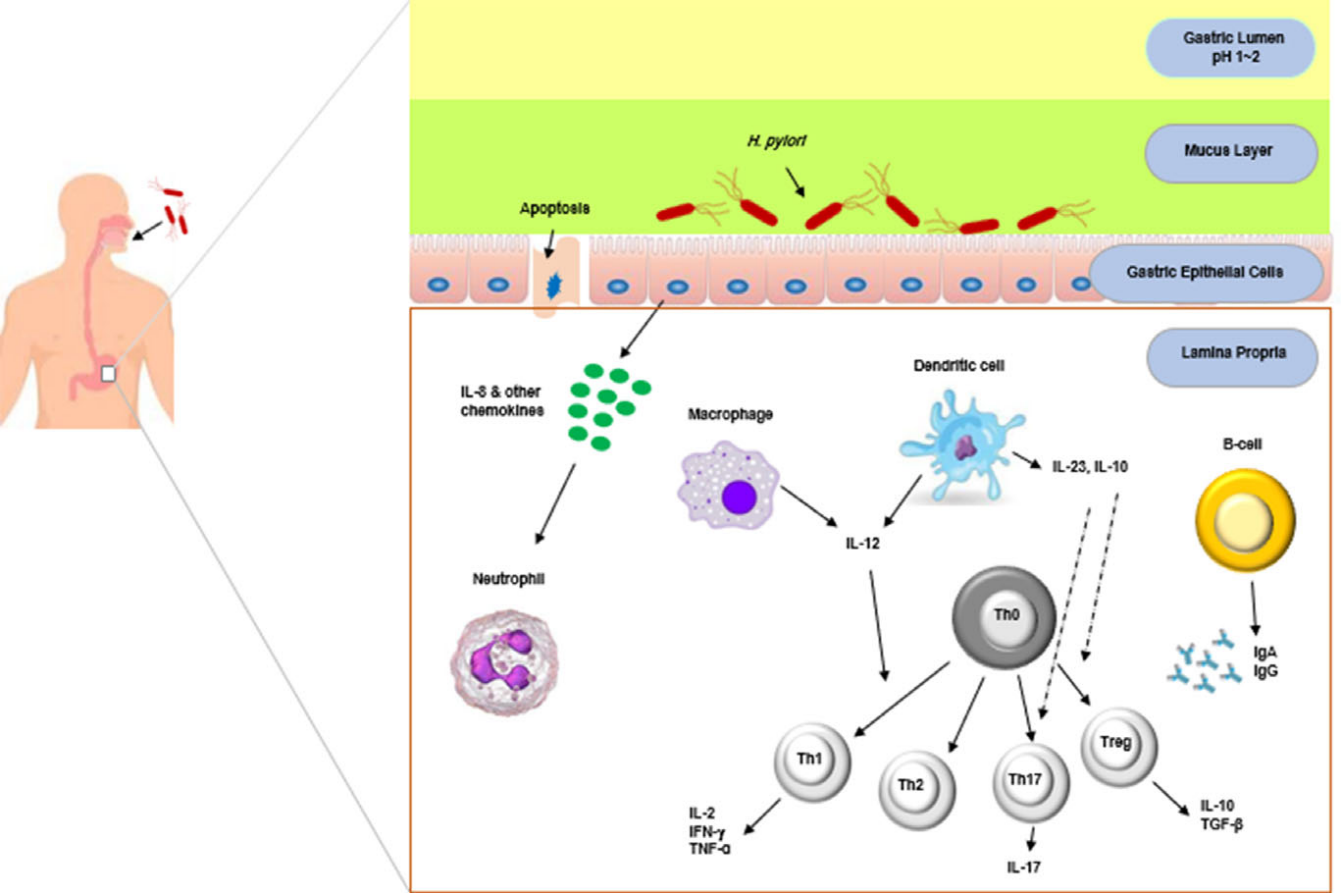
Schematic representation of the host immune system’s reactions to the *Helicobacter pylori* infection in the stomach. The first inflammation eradicates the bacteria and inhibits its dissemination. Capillary wall cells generate chemical mediators that infiltrate white blood cells at the site of injury during inflammation. As a result, neutrophils and monocytes in the blood are rejected. Dendritic cells, macrophages, neutrophils, lymphocytes and endothelium activate simple CD4^+^ T cells and trigger antigen-specific responses in Th1 and Th17 cells. Th1 cells produce IFN-γ and regulate cellular immunity, whereas Th17 cells produce IL-17. IL-12 and IL-23 are also present in *H. pylori*-stimulated macrophages. A T-reg regulatory cellular response is also observed, which enhances immunity while suppressing Th1- and Th17-induced immunity by generating IL-10 and TGF-β.

Vaccine design against *H. pylori* varies between pediatric and adult populations (Ref. 78). Most infections typically arise during childhood and persist without receiving any treatment throughout a person’s lifetime. Children often do not show symptoms and develop an immunological response that promotes tolerance. This response involves T-regulatory cells and their products, as well as immunosuppressive cytokines, including IL-10 and TGF-β. In contrast, adults with *H. pylori* infection experience a primarily inflammatory immune response that includes Th1 and Th17 cells, as well as inflammatory cytokines like TNF-α, IFN-γ, IL-1, IL-6, IL-8 and IL-17. Infected children generally experience less stomach inflammation and peptic ulcer disease compared to adults. Different vaccines may be necessary for children and adults because of the variations in the immune responses to *H. pylori* colonisation. One could argue that adults benefit more from therapeutic vaccines and children from prophylactic ones. The innate and specific immune responses against *H. pylori* are summarised in Figure 1.

### Antigen screening

**Figure 2.**
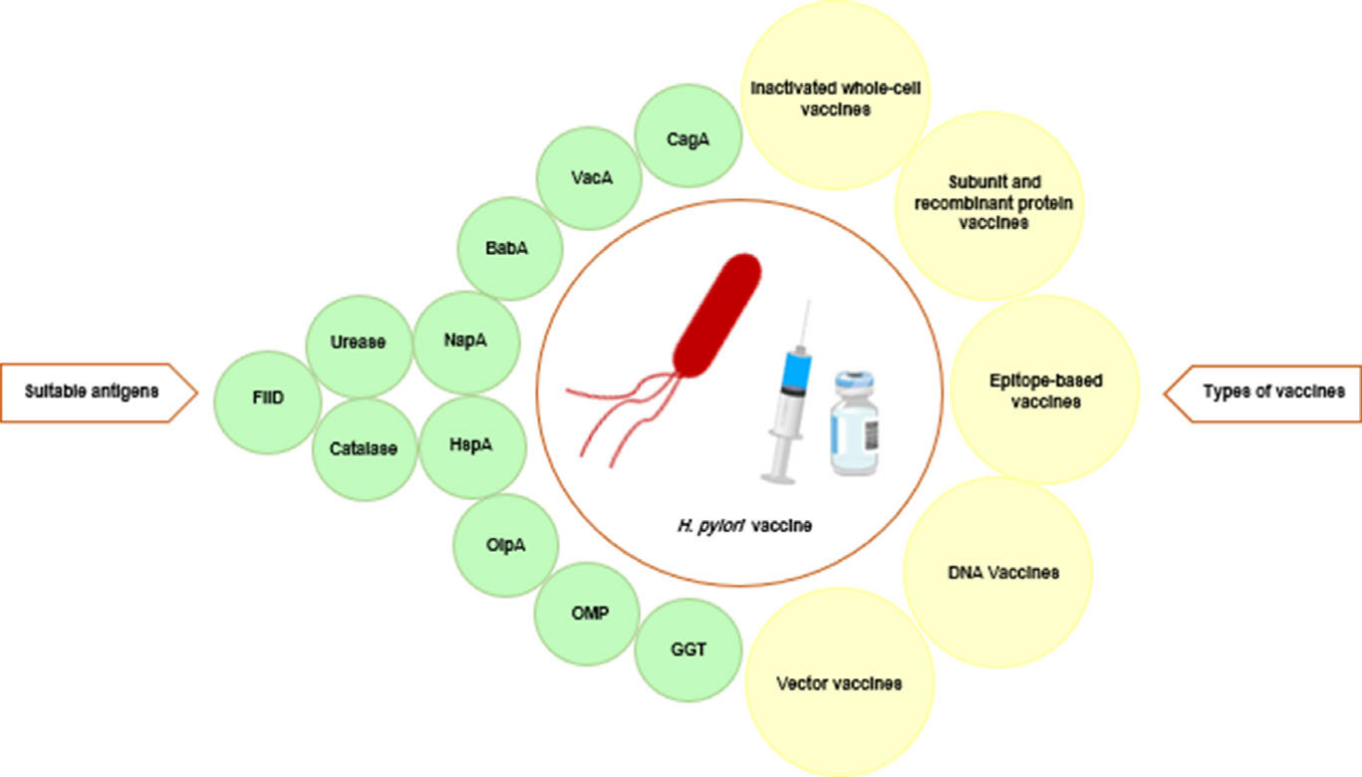
Most effective antigens and various types of vaccines used in vaccine development against *Helicobacter pylori.*

In order to prevent infections and/or treat existing diseases, vaccine-induced immunity must be achieved, which is known to be a complex process that depends on numerous variables. Considering the context of *H. pylori* infection, various antigens have been examined as prospective candidates for the development of vaccinations. It is widely acknowledged that vaccination antigens are often chosen based on unique traits. The presence of target antigens on the surface of the bacteria is necessary for their detection by the immune system. The antigens should be abundant, able to trigger an immune response, present in every bacterial isolate and factors that contribute to the pathogenicity of the bacteria (Refs. 19, 29, 79). Figure 2 shows a schematic representation of the primary targets for *H. pylori* vaccines that have been discussed in the literature. Some of these targets are described below.

#### cagPAI

The cag pathogenicity island (cagPAI) is a segment of the chromosome that spans 40 kilobases and contains a functional T4SS. This system is crucial for the development of *H. pylori*-related diseases. Within this region, there are three genes, namely, cagA, cagL and cagW, which can serve as potential antigens for incorporation into vaccines (Ref. 44, 80, 81). While the presence of cagPAI ensures the presence of a functional CagT4SS, around 30% of *H. pylori* strains lack cagPAI entirely, and in certain strains, it is only partially present (Refs. 82, 83). The clinical results caused by *H. pylori* vary in severity based on the presence of cagPAI. Consequently, partial deletions within cagPAI lead to a decrease in pathogenic characteristics (Refs. 84, 85). The cagPAI is present in around 70% of all *H. pylori* strains worldwide, with a prevalence of 60% in western isolates and 95% in East Asian isolates (Ref. 86).

The CagA is situated near the terminal region of cagPAI, which is strongly associated with the synthesis of VacA (Refs. 87, 88). Evidence suggests that CagA fragments can elicit an immune response. The recombinant protein CagA (rCagA) is bound to human antiserum (Ref. 89). Mohabati-Mobarez *et al.* showed that the combined-immunisation group of mice showed a robust Th1 immunoresponse, following rCagA and LPS immunisation, in contrast to the control group (Ref. 90). Paydarnia *et al.* also postulated that a CpG adjuvant containing *H. pylori* LPS and rCagA protein would generate a robust Th1-biased immunoresponse while also maintaining the recombinant protein’s antigenicity throughout the experiment (Ref. 91). Research indicates that CagA-positive strains have a greater ability to enhance the immune system’s function by activating dendritic cells and promoting the production of IL-12, IL-17 and IL-23. Therefore, this molecule is proposed as a potential antigen for enhancing vaccinations (Refs. 92–94). In addition, clinical trials have also shown that CagA is an excellent candidate antigen for eliciting immune responses (Refs. 30, 51).

Both CagW and CagL are proteins involved in the T4SS of *H. pylori* (Refs. 95, 96). CagA is able to travel past the bacterial membrane barrier as a result of the interaction with CagW, which offers favorable circumstances (Ref. 96). The use of cagW as a DNA vaccine resulted in significant activation of both the mucosal and humoral immune responses in mice (Ref. 44). CagL attaches to receptors on host cells and initiates the activation of signaling pathways (Ref. 97). Mice that have been immunised with recombinant cagL can make IgA antibodies that specifically target cagL (Ref. 80).

#### VacA

All strains of *H. pylori* have a single copy of the vacA gene on the chromosome, but only about half of these strains can make cytotoxin proteins (Ref. 98). VacA, which is associated with gastritis and peptic ulcers, induces cellular injury and the formation of pores in the plasma membrane (Ref. 99). *H. pylori*’s lifelong colonisation and pathogenesis are facilitated by VacA’s effects on host cells, which include induction of apoptosis, autophagy, membrane depolarisation, activation of MAP kinases, inhibition of T cell function, interfering with MHC II antigen presentation and mitochondrial dysfunction (Refs. 98, 100–105). Guo *et al.* recently developed a vaccine called FVpE employing a polysaccharide adjuvant (PA) that contains *Lycium barbarum* polysaccharides (LBPs) and chitosan. This vaccine has Th1 immunoadjuvants NAP, VacA, CagA and functional fragments of urease multiepitope peptides. When compared to the natural urease vaccine, FVpE is capable of eliciting elevated levels of antibodies that specifically target the antigen. Additionally, FVpE is able to significantly decrease the population of *H. pylori* in mice that are infected (Ref. 48). In phase II clinical research, a vaccination containing VacA, CagA and HP-NAP, along with aluminum hydroxide, induced targeted antibody and T cell responses to all three antigens in healthy volunteers who were negative for *H. pylori.* Compared to the placebo group, this vaccine can boost the immune system’s response to important *H. pylori* antigens. These antigens have been shown to be good candidates for vaccination because they contain vacuolating toxins (Ref. 30).

#### Urease

The production of urease by *H. pylori* is crucial for the bacterium’s ability to colonise and survive, leading to gastric infection (Ref. 57). The *H. pylori* urease is composed of UreB and UreA heterodimers, which together form a polyenzyme. This enzyme makes up approximately 10–15% of the total protein content in the bacteria (Ref. 106). The urease enzyme facilitates the transformation of urea into ammonia and carbon dioxide, which in turn elevates the acidic pH of the stomach to a neutral level. This process effectively neutralises the acidic environment, providing protection to *H. pylori* bacteria against its detrimental effects (Ref. 107). Carbon dioxide can shield bacteria from the poisonous effects of ONOO^−^, hence facilitating the growth and establishment of harmful microorganisms (Ref. 108). Ammonia has the ability to counteract excessive gastric acid, hinder the activity of neutrophils, facilitate the creation of harmful chemicals (Ref. 109) and disrupt the integrity of connections between gastric epithelial cells (Ref. 110). Inhibiting urease activity plays a role in preventing and treating *H. pylori* by limiting its ability to colonise the stomach (Ref. 111). Urease has been predominantly employed as a possible antigen in most research studies (Refs. 31, 66, 112–114). In a mouse model that has been infected with *H. pylori*, the administration of the genetically engineered plasmid pcDNA3.1 (+)-*ureA* can induce an immune response (Ref. 115). The urease antigen is found in most immunisations that have progressed to the clinical trial stage (Refs. 20, 50, 116–118).

#### Outer membrane proteins

*H. pylori* OMPs maintain the outer membrane structure, transfer materials and facilitate interaction with the host (Ref. 119). *H. pylori* OMPs are mostly lipoproteins, porins, iron-regulated proteins, efflux pump proteins and adhesins (Ref. 120). These OMPs can cause disease in three ways: by adhering to surfaces as adhesins, by breaking down protective barriers and by evading the immune system (Ref. 121). The adhesins of OMPs can activate the immunological response of the host cell and facilitate the intracellular transmission of signals in proinflammatory cells, thereby making OMPs suitable for use as an immunising antigen (Ref. 122).

*H. pylori* OipA is a key virulence component that helps bacteria adhere to host cells, resulting in the generation of proinflammatory cytokines and host adaptation (Refs. 123, 124). The OipA gene can be “on/off” as well. OipA production usually produces positive CagA, indicating that these two proteins are linked (Ref. 125). Chen *et al.* demonstrated that oral therapeutic immunisation with the Salmonella-delivered codon-optimised oipA construct (SL7207/poipA-opt) effectively eradicated *H. pylori* colonisation in the stomach in mice. Furthermore, protection was associated with a robust Th1/Th2 immune response (Ref. 126). In another study, Soudi *et al.* demonstrated that recombinant OipA, when administered orally or intravenously, can stimulate Th1 immunoresponse and generate IFN-γ production in mice (Ref. 127).

Blood-group antigen-binding adhesin (BabA) and sialic acid-binding adhesin (SabA) are the main types of adhesins that are needed for infection and colonisation. The BabA protein binds to fucosylated H-type 1 and Lewis B glycans, and the SabA protein recognises sialyl-Lewis A and X glycans (Ref. 128). Positive BabA in *H. pylori* strains is linked to duodenal ulcers and gastric adenocarcinoma progression, aiding in vaccine development (Ref. 129). SabA-expressing strains can cause gastric illnesses, excessive neutrophil infiltration and gastric atrophy after infection and have a high colonisation capacity (Ref. 130). Bugaytsova *et al.* found that administering the BabA vaccine to humans and rhesus macaques produced blocking antibodies, which reduced inflammation in the gastric mucosa, maintained gastric juice acidity and provided complete protection against *H. pylori*-induced gastric cancer in a mouse model (Ref. 131).

*H. pylori* adhesion A (HpaA) is a conserved lipoprotein that binds to glycosylated components on gastric epithelial cells, allowing *H. pylori* to attach to the mucosa (Refs. 132, 133). It also plays a role in dendritic cell development and antigen presentation (Ref. 133). The activation of TLR2 by HpaA depends on its N-terminal lipid component (Ref. 134). Tobias *et al.* found that administering formaldehyde-inactivated *Vibrio cholera*-expressing HpaA to mice increased serum antibody responses against HpaA, especially when co-expressed with fimbrial enterotoxigenic *E. coli* colonisation factors on the bacterial surface (Ref. 135).

#### Catalase

Catalase (CAT) breaks down hydrogen peroxide into water and oxygen, protecting the body from gastric acidity (Ref. 94). Its selection for anti-*H. pylori* vaccines is based on its significant expression rates (1% of the total protein of *H. pylori*) during pathogenic infection and its presence in various bacterial cell locations (Ref. 136). CAT protects bacteria from reactive oxygen species (Ref. 137) and macrophage engulfment (Ref. 138), acting as a defense mechanism against harmful effects from the host. Recently, CAT’s immunodominant Th1 epitopes were fully identified. Seven unique CAT epitopes promote a significant Th1 response via IFN-γ expression (Ref. 139). Miyashita *et al.* proved that immunisation with pcDNA3.1-*kat* by intranasal and intracutaneous routes can elicit substantial production of IgG antibodies, diminishing the severity of gastritis and effectively shielding mice from *H. pylori* colonisation (Ref. 140).

#### NAP

*H. pylori* NAP is an adhesion and is present in almost all *H. pylori* isolates. NAP preferentially attaches to high-molecular-weight mucins to help bind to host cells. NAP’s proinflammatory and immunomodulatory capabilities contribute to *H. pylori*-related diseases (Refs. 141, 142). Recent advances have been made in NAP’s potential as a vaccine candidate (Refs. 28, 48, 51, 143, 144). Scientists used a brand-new type of salmonella vaccine called PIESV to deliver and activate several *H. pylori* antigen genes. These genes are HpaA, Hp-NAP, UreA and UreB. In 70% of mice, this method completely prevented *H. pylori* SS1 infection. More IgG1, IgG2c, total IgG and stomach IgA antibodies were found in immunised mice than in control mice, and the immunised mice also had unique cellular memory responses (Ref. 145). In another study, mice administered with a multivalent subunit vaccine containing NAP, UreA, UreB and double-mutant heat-labile toxin as an adjuvant exhibited a notable immune response characterised by Th1/Th17 cell activation and the production of antigen-specific antibodies (Refs. 144, 146).

#### HspA

HspA, which is found in both the cytoplasm and on the cell surface (Ref. 61), has been identified as a suitable antigenic option for developing vaccines against *H. pylori.* HspA plays a crucial role in sequestering nickel for urease activity. Intranasal immunisation of mice with HspA resulted in decreased bacterial colonisation in the stomach. The protection was achieved through a robust immune response, both at the systemic and localised levels, involving the production of antibodies and a well-regulated balance of Th1/Th2 cytokines (Ref. 147). Zhang *et al*. discovered two immunogenic, highly conserved HspA B-cell epitopes (Ref. 148).

#### Lpp20

Lipoprotein 20 (Lpp20), a membrane-associated conserved lipoprotein, is only detected in *H. pylori.* Nearly, all *H. pylori* strains have Lpp20. Numerous studies have identified it as a promising *H. pylori* vaccine candidate due to its immunogenicity (Refs. 26, 149–151). Sun *et al.* successfully developed Lpp20 in *Lactococcus lactis* recombinants. This vaccine increased blood IgG and decreased gastric urease activity in mice when orally administered (Ref. 151). An *H. pylori* vaccine, based on a baculovirus, was administered through different routes. The Thp1 transgene in this vaccine codes for nine *H. pylori* epitopes. These are carbonic anhydrase, urease B subunit, GGT, Lpp20, Cag7 and CagL. The results showed a robust IgG-antibody response in the serum of mice, which was not dependent on the use of an adjuvant (Ref. 152).

#### GGT

GGT converts glutamine to glutamate and ammonia and glutathione to glutamate and cysteinyl glycine (Ref. 153). GGT functions in immune system activation by suppressing dendritic cell maturation, increasing Treg responses and altering the CD4^+^ T cell cycle, making it a viable vaccine target (Ref. 154). GGT-containing vaccinations block GGT rather than neutralising *H. pylori*, unlike other immune stimulants. This inhibition prevents T cell repression by increasing activated T cells and protecting against *H. pylori* infections (Ref. 155). Intranasal GGT and HspA immunisation reduced stomach bacterial colonisation in mice. Strong antibodies and a finely balanced Th1/Th2 cytokine response provided protection (Ref. 147).

#### Flagellin

Flagella, essential for bacterial motility, is required for *H. pylori* infection and colonisation. FlaA and FlaB components are crucial for gastric mucosal damage and could be potential antigens for vaccine development (Ref. 156). Mice were given a DNA vaccine, and the pBudCE4.1-*flaA* construct successfully expressed flaA in cells and raised levels of cytokines and immunoglobulins in their blood (Ref. 43). Yan *et al.* constructed the recombinant plasmid pET32a-*flaB* and showed that rFlaB has satisfactory immunoreactivity and antigenicity in mice (Ref. 157).

#### Multivalent and/or multiepitope vaccine

Individual subunit vaccines have limitations, including not providing immunity against all *H. pylori* antigens, not stimulating protective immune responses against different strains and potentially causing adverse reactions, such as allergic reactions or autoimmune diseases (Refs. 14, 29, 158, 159). In addition, existing *H. pylori* vaccines struggle due to the bacteria’s genetic variability. Also, *H. pylori* can adapt and evade the host’s immune response, making it difficult to develop a monovalent universal vaccination that targets all strains. The persistence of *H. pylori* infection requires a prolonged immune response, which is difficult to achieve with conventional vaccines (Refs. 160, 161). These issues highlight the need for novel vaccines that can overcome *H. pylori*’s genetic diversity. Creating a multivalent and/or multiepitope vaccination that targets multiple bacterium strains may increase the likelihood of immunity (Refs. 28, 48, 162).

As shown in Figure 2, the immunodominant antigens of *H. pylori* that elicit an immune response have been utilised in several forms of vaccines, including whole-cell vaccines (Ref. 163), DNA vaccines (Refs. 41, 44, 115, 126), subunit vaccines (Ref. 89, 131), vector vaccines (Refs. 80, 143, 150) and epitope-based vaccinations (Refs. 26, 28, 152).

### Genetic diversity

*H. pylori*’s high mutation and recombination rates create a diverse and ever-changing population within hosts, making vaccine development difficult (Ref. 164). This population’s genetic diversity can lead to specialised adaptations and strong natural selection, underscoring the necessity for a vaccination that targets this varied group (Refs. 164, 165). Immunogen virulence factors, including VacA and CagA, are generally targeted for *H. pylori* vaccination. However, these traits show genetic variability, complicating vaccine development (Ref. 166). To address this issue, a vaccination based on conserved epitopes that target many *H. pylori* proteins could be cost-effective and cover the bacteria’s genetic heterogeneity (Ref. 165). Innovative vaccination research uses immunoinformatics to locate T- and B-cell epitopes (Refs. 165–168). The development of a multivalent epitope-based vaccine aims to capture the genetic diversity of the bacterial population, resulting in long-lasting and efficient immune protection (Ref. 165).

### Choice of vaccine adjuvant

**Figure 3.**
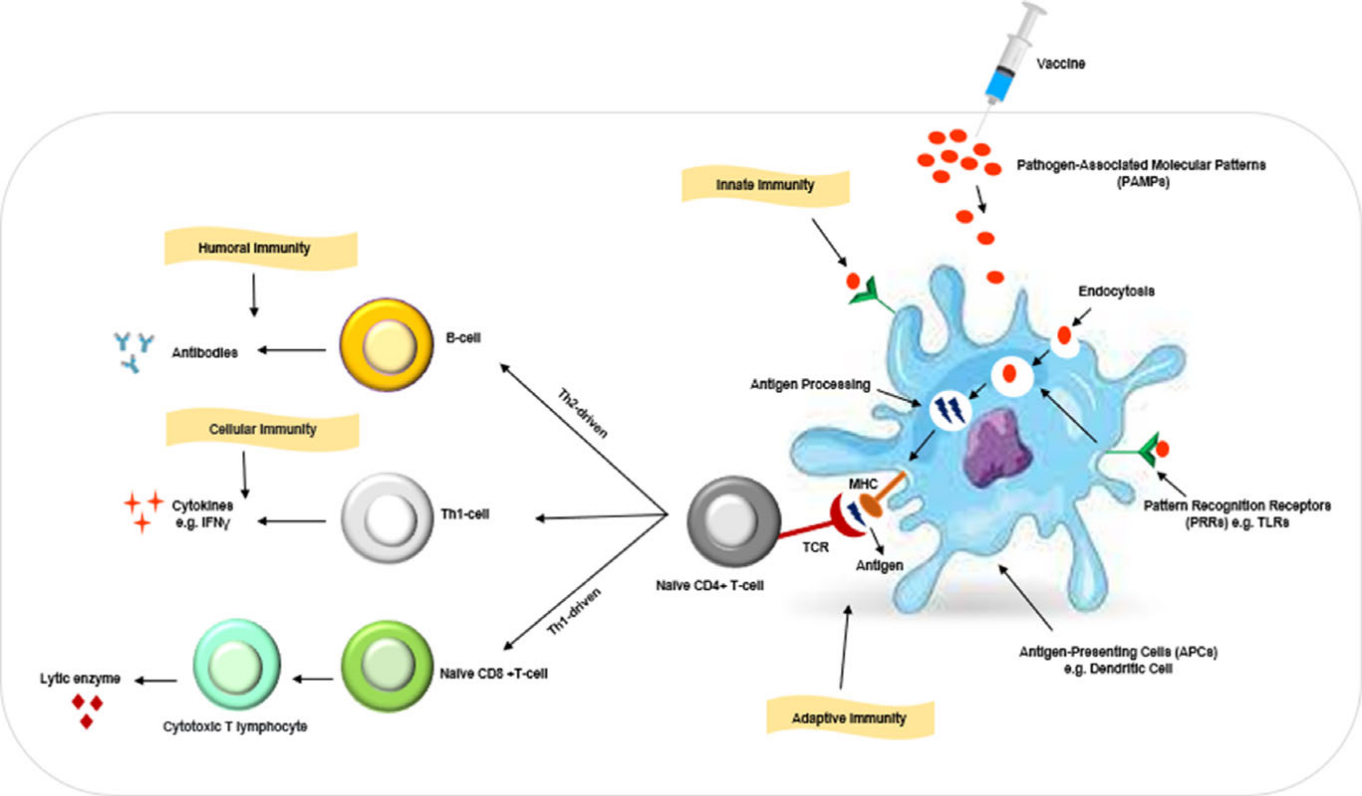
Overview of the function of vaccines and adjuvants. Antigenic proteins in vaccines, called pathogen-related molecular patterns (PAMPs), are presented to antigen-presenting cells (APCs) and are identified by their pattern recognition receptors (PRRs), such as toll-like receptors, at their surface. Adjuvants often act as PAMPs, which are identified by the PRR of the innate immune system. In the absence of adjuvants, mucosal delivery of vaccine antigens may result in T and B cell tolerance rather than effective immunization. Once identified, they are processed and placed on the major histocompatibility complex proteins (MHC-I or MHC-II) and are delivered to T cells native CD4^+^ that stimulate cellular and humoral immune responses. This stimulation leads to the production of antibodies in the humoral immune system and cytokines in the cellular immune system.

*H. pylori* proteins have limited immune response capabilities, making it difficult to eradicate the infection. Therefore, immunological adjuvants are essential during *H. pylori* vaccination. Adjuvants enhance the immune response’s potency and duration, alter the immunological response’s nature and reduce vaccine production costs by reducing the amount of immunogen used (Ref. 37). Also, adjuvants increase antigen immunity by enhancing inflammation and phagocytic penetration (Figure 3). The challenge lies in designing an adjuvant system for *H. pylori* vaccination, as existing efficacy in mice does not translate to humans, necessitating further experimentation and study to determine their suitability for human use.

#### Mutants of CTB and LTB

*E. coli* (ETEC) produces heat-labile enterotoxin (LT), a diarrhea-inducing toxin linked to CT (Ref. 169). Many studies have tried to make recombinants or mutants of CT or LT to lower their toxicity, even though they are very harmful to the intestines and cause severe side effects (Refs. 170–172). CT complexly regulates lymphokine generation, T cell proliferation, antigen presentation, IgA synthesis and B cell isotype differentiation. Its nontoxic binding subunit fraction (CTB) boosts mucosal immune responses to linked foreign antigens or epitopes (Ref. 26, 28, 173). Recently, Guo *et al.* constructed a multivalent epitope vaccine called FVpE, which includes the NAP, fragments from CagA and VacA and a urease epitope. This vaccine was found to enhance the protective effect of an oral vaccine by exacerbating mucosal inflammatory injury and inducing mixed CD4^+^ T cell responses (Ref. 48). There is strong evidence that vaccines with LTB as an immunoadjuvant can boost immunity (Refs. 133, 174, 175). LTB has some side effects but is used as an immunoadjuvant in most *H. pylori* vaccination clinical trials (Refs. 20, 41, 112, 118). In a clinical trial, Banerjee *et al.* demonstrated that low-dose LTB maintains immunogenicity and decreases toxicity (Ref. 116).

#### Cytokines

ILs are used as immune adjuvants in *H. pylori* vaccine development due to their ability to provide immunomodulatory effects at low doses through high-affinity specific receptors. Many studies have demonstrated that the DNA vaccination can preferentially elicit Th1 immunoresponse, including IL-2, IL-1, IL-6, IL-15 and IL-12, when combined with a cytokine gene-encoding plasmid (Refs. 45, 47, 176). IL-18, IL-17A and IL-22 modulate the immune response and enhance the efficacy of DNA vaccines. The co-administration of the OipA gene and IL-17A has been demonstrated to induce sterile immunity in mice challenged with *H. pylori* (Ref. 45). Another study inoculated mice mucosally with the recombinant *Lactobacillus lactis*-expressing UreB-IL-2 chimeric protein. This vaccine produced anti-UreB antibodies, lowered the bacterial load and elevated IFN- γ, IL-4 and IL-1 (Ref. 176).

#### Chitosan

The utilisation of chitosan, a natural polysaccharide derived from D-glucosamine and chitin, as an adjuvant in a *H. pylori* vaccine has been investigated in the studies conducted by Gong *et al.* and Xie *et al.* Chitosan, characterised by its non-toxicity, non-irritability, non-allergenicity, biodegradability, biocompatibility and bioadhesiveness, has shown promising results in these studies. Gong *et al.* reported that a chitosan-adjuvanted *H. pylori* vaccine elicited higher levels of *H. pylori*-specific antibodies and cytokines, including IFN-γ, IL-10, IL-2 and IL-12, and achieved a superior *H. pylori* elimination rate of 58.33%, compared to a CT-adjuvanted vaccine with an elimination rate of 45.45% (Ref. 39). Furthermore, Xie *et al.* found that the chitosan-adjuvanted vaccination generated both Th1 and Th2 immune responses and gave immunoprotection in 60% of the tested mice, a substantially greater rate than that observed in the *H. pylori* antigen-only group (Ref. 42). These findings underscore the potential of chitosan as an efficacious adjuvant in *H. pylori* vaccination.

#### cGAMP

Cyclic guanosine monophosphate-adenosine monophosphate (cGAMP) is a signaling molecule that regulates the body’s immune responses and enhances antigen-specific responses, particularly the Th1 response (Ref. 177). It is created when DNA ligands stimulate cyclase, activating the STING receptor protein and producing cytokines (Ref. 178). STING agonists like cGAMP are promising immunoadjuvants (Ref. 179). Chen *et al.* found that intranasal and subcutaneous vaccinations with recombinant *H. pylori* UreA, UreB and NAP adjuvanted with cGAMP reduced stomach mucosal colonisation in mice. Antigen-specific serum IgG and mucosal IgA responses increased considerably in all challenged immunised animals. Only intranasally infected mice produced IL-17 responses, which were connected to antigen-specific Th1 and Th17 responses and vaccine-induced protection (Ref. 180).

#### CpG ODNs

TLR 9 can recognise CpG oligodeoxynucleotides (CpG ODNs), which turn on immune cells and are added to vaccines to protect against cancer, allergies and infections (Refs. 181–183). Studies have shown their effectiveness in eliciting immune responses against *H. pylori* in mice, with intranasal administration of CpG ODNs with whole cell antigens, significantly increasing specific IgG, IgA and IFN-γ responses and enhancing protection against infection (Ref. 40, 184). Furthermore, the combination of the rCagA protein with CpG not only maintains the antigenicity of the recombinant protein but also stimulates a strong immune response, specifically targeting Th1 cells (Ref. 91). These findings underscore the potential of CpG ODNs as effective mucosal adjuvants for *H. pylori* vaccines.

#### α-GalCer

α-Galactosylceramide (α-GalCer) is a glycolipid obtained from a marine sponge that triggers both humoral and cellular immune responses (Ref. 185). It activates iNKT cells through CD1d, resulting in the release of Th1 and Th2 cytokines (Refs. 186, 187). The impact of the α-GalCer adjuvant closely resembles that of conventional CTB (Ref. 21). α-GalCer as an adjuvant can enhance immune responses to various pathogens, including *H. pylori*, the *herpes simplex virus* and enterotoxin-producing *E. coli* (Refs. 21, 188, 189). In the case of *H. pylori*, relying on the signaling of CD1d, IL-1R and IL-17R, intragastric immunisation against *H. pylori* using whole-cell inactivated antigen and α-GalCer produced strong Th1 cellular immune responses and antigen-specific antibody responses in both mucosal and systemic regions (Ref. 21). Overall, α-GalCer shows promise as an adjuvant for oral vaccinations targeting *H. pylori* infection, as it enhances immune responses and promotes protective mucosal immunity.

#### Plant polysaccharides

Plant polysaccharides, such as Astragalus polysaccharides, Epimedium polysaccharides, chitosan and LBPs, are biologically active compounds that possess distinctive properties and minimal toxicity (Ref. 190). Studies have demonstrated that PAs are efficacious vaccination adjuvants that enhance both cellular and humoral immunity (Refs. 191–193). For instance, the addition of chitosan and polysaccharide mucosal adjuvant in LBPs has been found to improve the efficacy of the protective effect of a multivalent epitope (CagA, VacA and NAP) vaccination (Ref. 48). Similarly, the Astragalus polysaccharides and rUreB can stimulate a combined Th1 and Th17 immune response, potentially enhancing the mice’s ability to defend against *H. pylori* infection (Ref. 194).

#### Propolis

Propolis is a resinous compound collected by honeybees from flowers and has immunostimulatory and immunomodulatory properties (Ref. 195). In a study, the use of propolis as an adjuvant with an inactivated vaccine against swine herpesvirus type 1 (SuHV-1) resulted in increased cellular and humoral immune responses compared to a control vaccine (Ref. 196). Another study found that propolis as an adjuvant increased the level of IFN-γ by increasing the mRNA synthesis of IFN-γ and enhanced the intensity of the cellular immune response in mice vaccinated with an *H. pylori* OipA protein vaccine (Ref. 127). This suggests that propolis, as an adjuvant, can contribute to the effectiveness of vaccines.

#### Melittin

Melittin, the primary constituent of bee venom, is composed of 26 amino acids and possesses immunomodulatory properties that augment the production of IFN-γ and thus boost the functionality of Th1 cells. This brief peptide also has the capacity to decrease IL-10 and enhance IL-1β in the equilibrium of cytokines. Melittin can serve as an adjuvant for the *H. pylori* vaccination. Jafari *et al.* designed, produced and isolated a multiepitope vaccine comprising CD4^+^ T cell epitopes of UreB, HpaA and NapA antigens, with an emphasis on IFN-γ production targeting *H. pylori*, utilising melittin as an adjuvant. However, the efficacy of using melittin as an adjuvant in the *H. pylori* vaccine has not been documented.

### Vaccine delivery systems

Developing a safe and effective vaccine against *H. pylori* is crucial for eradicating the bacterium on a large scale. However, the complexity of the mucosal immune environment has made this challenging (Ref. 23). These systems aim to enhance the immune response by delivering antigens in a targeted and efficient manner. The choice of the delivery system depends on factors such as the target antigen, desired immune response and specific vaccine application (Ref. 197). Each system has its own advantages and can contribute to the development of safe and effective *H. pylori* vaccines. Despite the development of various adjuvants and delivery modalities for immunisation, there is currently no licensed inactivated whole cell vaccination for *H. pylori.* Enhancing the immunogenicity and ensuring the safety of vaccines continue to be challenges (Ref. 36).

#### OMVs

Outer membrane vesicles (OMVs), which contain proteins, poisons and lipids, play a significant role in bacteria–host interactions (Ref. 198). They have shown promise as a delivery mechanism for antigens with the successful transportation of heterologous proteins to vesicles (Ref. 199). Two articles discuss the potential of OMVs as delivery systems to promote protective efficacy against *H. pylori* infection in mice. Song *et al.* found that orally administered OMVs from *H. pylori* 7.13 showed protective activity without significant toxicity. OMVs triggered Th2-based immune responses, reducing the bacterial load after *H. pylori* Sydney strain 1 assault. Liu *et al.* demonstrated that OMVs reduced *H. pylori* infection via Th2-biased immune responses (Ref. 200). Moreover, OMVs are recognised as a promising adjuvant because of their minimal toxicity and capacity to elicit a comprehensive immune response (Ref. 201).

#### Vaccine vectors

The research articles offer useful insights on the prospective utilisation of bacterial, yeast and viral vectors for the advancement of vaccines against *H. pylori* infection (Ref. 36). The attenuated vector can display *H. pylori* immunogens to antigen-presenting cells, activating host immune responses. Hence, vector vaccines mimic natural infection, causing a lasting immune response (Refs. 33, 145).

#### Bacteria

The mucosal delivery of lactic acid bacteria target proteins can trigger systemic humoral and cellular immunoresponses (Ref. 202). Gou *et al.* created LL-plSAM-FVpE, an *L. lactis* surface display method targeting M cells. plSAM can increase M cell phagocytosis and transport of antigens in the gastrointestinal tract and elicit a protective immunoresponse (Ref. 32). In another study, high mucosal SIgA antibody levels and enhanced mouse protection against *H. pylori* infection can be achieved with recombinant *Lactobacillus acidophilus* expressing Hp0410 (Ref. 203). A *L. lactis* strain was used to express HpaA and Omp22, and the orally vaccinated mice had a strong systemic humoral immune response compared to PBS controls (Ref. 204). Aliramaei *et al.* created a *L. lactis* MG1363-carrying CagL vaccine, and the levels of specific IgA, IL-17 and IFN-γ dramatically increased in mice (Ref. 80). *L. lactis*-delivering Lpp20 effectively reduces the bacterial load in *H. pylori*-challenged mice. The serum IgG levels and lowered urease activity in the stomach following *H. pylori* challenges demonstrated its potential for mucosal immunisation against *H. pylori* (Ref. 151).

Live immunisation with attenuated Salmonella can induce an immune response against Salmonella and stimulate mucosal, humoral and cellular immunity to transport antigens after immunisation (Ref. 205). Nasal immunisation of mice with *Salmonella typhimurium* phoPc-expressing *H. pylori* urease A and B subunits made 60% of mice resistant. This shows that the vaccine can induce Th1- and Th2-type responses, protecting against *H. pylori* (Ref. 206). Chen *et al.* developed an attenuated *S. typhimurium* bacterial ghost (SL7207-BG) vaccination to deliver an *H. pylori OipA* gene DNA vaccine. This immunisation reduced bacterial colonisation in C57BL/6 mice challenged with *H. pylori* strain SS1 and elicited a mixed Th1/Th2 immune response (Ref. 207). T cell reactivity against *H. pylori* antigens was linked with the elimination or considerable reduction of *H. pylori* burden in volunteers who were orally inoculated with *Salmonella enterica* serovar Typhi Ty21a, producing *H. pylori* urease (Ref. 50). Oral administration of a live, attenuated *S. enterica* serovar Typhi vaccine generated mucosa-homing CD4^+^ and CD8^+^ T lymphocytes. These immune-enhancing cells may target *H. pylori*’s habitat (Ref. 208). These studies collectively suggest that Salmonella-based vaccines can induce protective immunity against *H. pylori* infection, potentially offering a promising strategy for controlling this common bacterial infection.

Researchers used *Bacillus subtilis* spores to deliver *H. pylori* urease B, using the spore coat protein CotC as a fusion partner. The result showed significant levels of urease B-specific IgA and IgG in feces and serum, indicating an immune response. Spore-carrying CotC-UreB was administered orally to a mouse model, resulting in an 84% reduction in *H. pylori*-positive mice (Ref. 209). Recently, a vaccine based on spores of *B. subtilis*- and *H. pylori*-protective antigens UreA and UreB has shown potential for further development and clinical trials. Mice were orally inoculated and challenged with *H. pylori* to assess immunological responses and colonisation. Antigen-specific mucosal responses (fecal sIgA), seroconversion (serum IgG) and up to 1-log less *H. pylori* load indicate the development of protective immunity (Ref. 210).

The Shigella 2aT32-based vaccination tested the UreB-HspA fusion antigen for *H. pylori* protection in mice. Oral administration with or without a parenteral boost produced specific antigen immune responses and dramatically reduced *H. pylori* colonisation after challenge, suggesting the vaccine’s ability to prevent *H. pylori* infection (Ref. 211).

The optimised attenuated *L. monocytogenes* carrying a multiepitope chimeric antigen can significantly reduce the colonisation of *H. pylori* and induce a high level of anti-*H. pylori* antibodies after intragastric and intravenous immunisation (Ref. 33).

#### Yeasts

Cen *et al.* developed a *Saccharomyces cerevisiae*-based oral vaccine, producing recombinant UreB and VacA. The vaccine demonstrated significant humoral and mucosal immunoresponses and significantly reduced the *H. pylori* load in mice (Ref. 212).

#### Viruses

It may be possible to improve long-lasting immunity against *H. pylori* by the use of viral vectors (Ref. 36). Clinical trials have demonstrated that the measles virus (MV) may offer a novel and flexible approach to the treatment of infectious diseases and cancer (Ref. 213). In a study, mice received a baculovirus containing a Thp1 transgene encoding nine *H. pylori* epitopes intramuscularly, intragastrically and intranasally. *H. pylori*-specific IgG and IgA antibodies were found in serum samples 125 days and feces samples 82 days after immunisation, respectively (Ref. 152). A recombinant MV Edmonston vaccination strain expressing the *H. pylori* HspA antigen was created by Iankov *et al.* The outcomes demonstrated the recombinant MV-HspA strain’s potent immunogenicity to the *H. pylori* HspA antigen, as well as its potent anticancer activity. To improve these viruses’ efficacy, safety and administration, more research is needed (Ref. 214).

#### Nanotechnology

Nanotechnology has the potential to boost *H. pylori* vaccine efficacy by limiting degradation and improving delivery. With current *H. pylori* treatment methods failing, developing a vaccine that can be distributed effectively could be a cost-effective solution to manage *H. pylori* epidemics (Ref. 215).

Zhang *et al.* developed a self-assembling nanoparticle with hydrophilic and slightly negative surface properties containing UreB, which demonstrated enhanced systemic and mucosal immune responses in mice, suggesting their potential as oral vaccines against *H. pylori* (Ref. 216). The researchers synthesised protein nanocapsules using the A subunit of *H. pylori* urease (UreA) and tested their efficacy in a mouse model. The study found that mice vaccinated with the nanocapsules, combined with an adjuvant andshowed significantly reduced *H. pylori* colonisation (Ref. 217). Liu *et al.* designed HP55/poly (n-butylcyanoacrylate) (PBCA) nanoparticles to carry the *H. pylori* subunit vaccine, CCF. The nanoparticles promoted the production of serum antigen-specific antibodies, mucosal secretory IgA and proinflammatory cytokines. In mice vaccinated with HP55/PBCA-CCF NP, stomach tissue showed an enhanced Th1/Th17 immune response and lymphocyte activity, possibly limiting *H. pylori* colonisation (Ref. 218). Additionally, Yang *et al.* developed an intranasal vaccine nanoemulsion containing a dominant HpaA epitope peptide. The system’s delayed antigen release elicited a significant Th1 immune response. The nanoemulsion prolonged the epitope peptide in the nasal cavity and boosted its absorption into cells, boosting vaccination-induced Th1 immune responses and reducing bacterial colonisation. Mixing the vaccine with a CpG adjuvant increased protection (Ref. 219). However, although nanoemulsions are widely used for combating bacterial growth and are easy to produce and preserve, there are very few studies on the eradication of *H. pylori* using them (Ref. 220). Therefore, the applicability of nanoemulsions as effective alternatives for *H. pylori* therapy requires further investigation. In summary, these studies highlight the potential of nanoparticle-based vaccines for combating *H. pylori* infection.

### Vaccine route administration

*H. pylori* vaccine administration routes struggle to produce a significant and protective immune response. The vaccine administration method affects immune response type and magnitude. Oral, nasal, parenteral, rectal, subcutaneous and intramuscular administration routes have all been investigated for the *H. pylori* vaccine. Kleanthous *et al.* studied UreA-LTB administration via oral, nasal and rectal routes in mice. All routes of administration prevented *H. pylori* infection and dramatically reduced stomach urease activity relative to the sham-immunised control group. All mouse immunisation strategies reduced *H. pylori* by 97%. Before the *H. pylori* challenge, rectal immunisation produced the most gastric antiurease IgA (Ref. 221). Another study investigated the protective effect of a multicomponent (UreB, HspA and HpaA) vaccine with two different adjuvants (Al (OH)_3_ and LT (R72DITH)) in administration either intragastrically or intramuscularly to Mongolian gerbils against *H. pylori* infection. The triple antigen vaccine combined with the LT (R72DITH) adjuvant showed an average protection rate of 86.3%, which was significantly higher than the vaccine combined with the Al (OH)_3_ adjuvant (average 53.4%) both intragastrically and intramuscularly. The intragastric route induced higher levels of gastric anti-*H. pylori* IgA and IgG and lower levels of gastric inflammation and ulceration compared with the intramuscular route (Ref. 222).

For *H. pylori*, mucosal immunity is particularly important, as the infection occurs in the gastric mucosa. Oral vaccines are attractive because they can directly target the mucosal immune system and are more convenient and acceptable, especially in low- and middle-income countries (LMICs), where the burden of *H. pylori*-related diseases is the highest (Ref. 223). Oral vaccines are a promising approach due to their direct action on mucosal immunity, but they must be designed to withstand the harsh gastrointestinal environment. The development of mucosal vaccines for *H. pylori* infection has faced several challenges, including the complexity of the host immune response, the lack of safe mucosal adjuvants and the inconsistent results obtained from different mucosal routes of vaccination, such as sublingual, rectal and intranasal (Refs. 21, 30, 224, 225). Also, the barrier provided by mucosal surfaces to prevent antigen delivery and immune response is the constant exposure of mucosal surfaces to commensals and innocuous foreign substances, which may lead to tolerogenic responses (Refs. 226–228). Moreover, the dose of mucosal vaccine that actually enters the body cannot be accurately measured due to the labor-intensive and technically challenging recovery and functional testing of mucosal T cells (Ref. 223). As a result, only a few mucosal vaccines have been approved for human use, and they were not specifically designed for mucosal application. Despite these challenges, some studies have shown promising results in using various adjuvants and antigens to induce protective immune responses (Ref. 21, 229). For example, an oral α-GalCer-adjuvanted *H. pylori* vaccine has been found to induce protective IL-1R- and IL-17R-dependent Th1 responses (Ref. 21). However, more research is needed to overcome the barriers associated with mucosal vaccination and to develop an effective *H. pylori* vaccine.

Intramuscular vaccines with adjuvants have shown efficacy in animal models, but more research is needed to optimise these vaccines for human use. Challenges associated with these routes of immunisation include the need to overcome the immune-modulating capacity of *H. pylori*, the development of resistance to treatment and the host’s propensity to downregulate the immune response following infection (Ref. 30). Some studies have explored the use of different adjuvants, such as aluminum hydroxide, to enhance the immune response to *H. pylori* antigens (Refs. 30, 224). However, no study has reported protective immunity with intramuscular vaccines (Ref. 230). However, the most promising route of administration for *H. pylori* vaccines in humans is yet to be conclusively determined and requires further research and development, as challenges such as the need to induce sterilising immunity and the selection of the right adjuvant for human use remain.

### Selection of animal models for vaccine evaluation

To test *H. pylori* preventive and therapeutic vaccinations, animal models must be colonised and given pathophysiological conditions that mimic human gastrointestinal illnesses (Ref. 231). Finding an acceptable model is challenging due to chronic stomach colonisation and unknown infection patterns (Ref. 16). The intricate interaction between *H. pylori* and the stomach epithelium over decades produces gastric cancer. Thus, animal models of *H. pylori* infection and immune response are being sought (Refs. 232,233). *H. pylori* may infect dogs, cats, pigs, monkeys, mice, Mongolian gerbils and guinea pigs (Ref. 16). Below, we delve into the top animal models.

*H. pylori* Sydney strain 1 causes gastric cancer and CG in mice, but wild-type models like BALB/c and C57BL/6 cause moderate gastritis or slowly progressing diseases (Refs. 234–236). These models provide limited insights into *H. pylori* pathogenicity, as the mouse stomach’s structural makeup differs from the human stomach and may include microorganisms affecting infection (Ref. 237, 238). To study *H. pylori*, several mouse models, including insulin–gastrin, IFN-γ, TNF-α, IL-1β and IL-10 knockouts, Fas antigen transgenic, p27-deficient and CagA-transgenic mice, are used (Ref. 231).

The most common animal model for *H. pylori* infection is Mongolian gerbils. Mongolian gerbils mimics human *H. pylori*-induced stomach colonisation, inflammation, ulceration and carcinogenesis (Refs. 239, 240). Several further studies have demonstrated that Mongolian gerbils exposed to *H. pylori* develops stomach, duodenal and intestinal metaplasia (Refs. 241–243). *H. pylori* colonisation of the stomach mucosa causes a varied lamina propria inflammatory infiltrate, similar to human diseases. This infiltration contains neutrophils and mononuclear leukocytes (Ref. 244, 245). Hence, they are effective and affordable rodent models.

Guinea pigs are lab animals with human-like stomachs. It can create an inflammatory response from stomach epithelial cell IL-8 release. Like the mouse model, guinea pig models show how easy animal care is due to their small size. The guinea pig stomach also has a cylindrical epithelium, maintains sterility, produces IL-8 and lacks a non-glandular area (Refs. 246, 247).

*H. pylori* strains can infect macaques (Ref. 248). Macaques may acquire *H. pylori* from humans or be a natural reservoir for the pathogen. Rhesus macaques offer many advantages over tiny animal models. Socially housed rhesus macaques are naturally infected with *H. pylori* and resemble humans physiologically and morphologically (Ref. 249). Additionally, all infected macaques will develop chronic gastritis, and a fraction may develop gastric atrophy, a histological characteristic that precedes gastric cancer (Ref. 250). However, studies on non-human primates are time-consuming, laborious and expensive, making it impossible to assess *H. pylori* pathogenicity. *H. pylori* typically infects the human stomach mucosa; however, few captivity-raised macaques are spontaneously infected (Ref. 251).

Finding an animal model that accurately replicates all features of *H. pylori* infection in humans is challenging. While mouse models provide limited insights into *H. pylori* pathogenicity, Mongolian gerbils are effective and affordable rodent models that mimic human *H. pylori*-induced stomach colonisation, inflammation, ulceration and carcinogenesis. Guinea pigs, with their human-like stomachs, can also create an inflammatory response similar to that of humans. Macaques offer advantages as they are naturally infected with *H. pylori* and resemble humans physiologically and morphologically, but studying them is time-consuming, laborious and expensive. Overall, based on our present understanding of virulence factors and their interactions with the immune system, it may be required to select an animal model based on certain optimum conditions. Factors such as the utilisation of antigens that activate cellular or humoral immunity, recruiting various cells of the immune system, and categorising the vaccine as therapeutic, prophylactic and anti-disease rather than anti-pathogen might play a crucial role in selecting the appropriate animal model. Thus, given the present circumstances, it may be unattainable to accomplish all required objectives with a solitary animal model.

## Conclusions and prospects

An optimal *H. pylori* vaccination for human use should possess not only efficacy and safety but also necessitate high patient adherence and provide durable protection over an extended period of time. Despite the efforts, an effective vaccine against *H. pylori* infection has not yet been developed (Ref. 37). The key challenges in designing vaccines against *H. pylori* include (1) the considerable genetic diversity and molecular mimicry exhibited by *H. pylori*; (2) the immune evasion strategies employed by *H. pylori*; (3) the constraints in choosing suitable animal models and (4) the identification of an appropriate vaccine delivery system to overcome various obstacles in the stomach. This review adds to the existing knowledge by summarising the advances in *H. pylori* vaccine research, including host–immune interaction, candidate antigens, adjuvants, animal models and delivery systems.

Several vaccine candidates have been explored, including recombinant subunit vaccines using UreB, VacA, CagA, NapA, HpaA and so on as the vaccine antigen, which have shown good prophylactic effects. Multiple investigations have shown that single-antigen immunity against *H. pylori* is insufficient. Immunity to *H. pylori* is typically provided by administering a cocktail of antigen subunits or combining epitopes from several antigens (Refs. 165, 167). Thus, many research institutions create *H. pylori* vaccines using various antigens. Epitope-based vaccines are cheaper than mixed proteins and can target more protein targets. Thus, multiepitope vaccinations are gaining interest (Refs. 19, 29, 48, 252). In this scenario, advanced contemporary immunoinformatic techniques can also be employed in the development of multiepitope vaccines (Refs. 253–255).

An effective *H. pylori* vaccine could substantially reduce the burden of bacterial load, gastric cancer and other *H. pylori*-related diseases, particularly in developing countries. Nevertheless, several endeavors have been made in preclinical and clinical trials to attain sterile immunity, following prophylactic or therapeutic vaccination against *H. pylori.* Perhaps, it is now opportune to shift our perspective towards an antidisease approach rather than an antibacterial one. Also, not everyone who is infected with *H. pylori* develops these diseases, and some studies suggest that *H. pylori* may also have some beneficial effects, such as protecting against asthma and inflammatory bowel disease (Refs. 256, 257). Therefore, some researchers are exploring the possibility of developing a vaccine that does not aim to eliminate *H. pylori* from the stomach but rather to modulate the immune response and reduce the harmful inflammation that it triggers (Ref. 258). Such a vaccine would target the specific molecular pathways that are involved in the inflammatory process and could potentially prevent or treat the diseases associated with *H. pylori* infection while preserving its possible benefits.

Future research could concentrate on (1) identifying immune responses related to protection in experimental models; (2) developing a better understanding of the protective mechanisms and identifying a cocktail of strong protective antigens or recombinant bacterial strains expressing such antigens; (3) investigating novel vaccine delivery methods and adjuvants to improve the effectiveness of *H. pylori* vaccines; (4) using mRNA vaccines capable of encoding many antigens and inducing both humoral and cellular protection; (5) creating multivalent vaccines that can target different strains and variants of *H. pylori*, as well as different stages of infection and disease progression, and (6) testing alternative immunisation routes that can elicit both systemic and mucosal immunity, such as intranasal, oral or sublingual administration.

Despite significant progress in *H. pylori* vaccine research, there is still a need for further advancements to develop an effective vaccine against this prevalent pathogen. Addressing the challenges and limitations associated with vaccine development, as well as fostering collaboration with industrial partners, could pave the way for the successful development of an *H. pylori* vaccine.
